# Breast Cancer Characterization Based on Image Classification of Tissue Sections Visualized under Low Magnification

**DOI:** 10.1155/2013/829461

**Published:** 2013-08-31

**Authors:** C. Loukas, S. Kostopoulos, A. Tanoglidi, D. Glotsos, C. Sfikas, D. Cavouras

**Affiliations:** ^1^Department of Medical Physics, Medical School, University of Athens, 75 Mikras Asias Street, 115 27 Athens, Greece; ^2^Medical Image and Signal Processing Laboratory, Department of Medical Instruments Technology, Technological Educational Institute of Athens, 12210 Athens, Greece; ^3^Department of Histopathology, Elena Venizelos Hospital, 106 72 Athens, Greece

## Abstract

Rapid assessment of tissue biopsies is a critical issue in modern histopathology. For breast cancer diagnosis, the shape of the nuclei and the architectural pattern of the tissue are evaluated under high and low magnifications, respectively. In this study, we focus on the development of a pattern classification system for the assessment of breast cancer images captured under low magnification (×10). Sixty-five regions of interest were selected from 60 images of breast cancer tissue sections. Texture analysis provided 30 textural features per image. Three different pattern recognition algorithms were employed (kNN, SVM, and PNN) for classifying the images into three malignancy grades: I–III. The classifiers were validated with leave-one-out (training) and cross-validation (testing) modes. The average discrimination efficiency of the kNN, SVM, and PNN classifiers in the training mode was close to 97%, 95%, and 97%, respectively, whereas in the test mode, the average classification accuracy achieved was 86%, 85%, and 90%, respectively. Assessment of breast cancer tissue sections could be applied in complex large-scale images using textural features and pattern classifiers. The proposed technique provides several benefits, such as speed of analysis and automation, and could potentially replace the laborious task of visual examination.

## 1. Introduction

Excluding skin cancer, breast cancer is the most common cancer among women, accounting for nearly 1 in 3 cancers diagnosed in US women. Currently, a woman living in the US has a 12.15% lifetime risk of being diagnosed with breast cancer, whereas in the 1970s this lifetime risk was less than 10%. In 2011, more than 200,000 women in the US were diagnosed with breast cancer [1], resulting in 40,000 deaths. In the past five years, the median age at the time of breast cancer diagnosis was 60 years, and 50% of women who developed breast cancer were younger than 60 years old at the time of diagnosis [2]. Postmenopausal obesity, use of combined estrogen and progestin menopausal hormones, alcohol consumption, and physical inactivity are some of the well-recognized risk factors of breast cancer by the National Cancer Institute [3].

While clinical assessment clues (breast examination or imaging results) may be strongly suggestive of a cancer diagnosis, microscopic analysis of breast tissue is necessary for a definitive diagnosis of breast cancer and to determine whether the cancer is *in situ* or invasive. The microscopic analysis can be obtained via a needle biopsy or a surgical biopsy. Selection of the type of biopsy is based on individual factors and availability. Numerous studies have attempted to improve the diagnosis of cancer, based on the analysis of cell images [4]. Since the early 1970s' cytology automation has been a major biomedical research field for the application of computer-assisted image analysis. Considerable effort has been devoted to the analysis of cellular images, particularly in the application areas of blood cell analysis [5] and cytology screening [6]. The overall effort and the degree of success have been restricted in a large part due to the simplicity of the images themselves, usually containing a few isolated cells against a plain background. Unlike cytological images, the structure of a histological microscopic section is usually much denser than that of the cytological one, since it reflects the structure of the entire tissue, and there is often a bewildering variety of touching and overlapping cells. The images are usually corrupted by noise and other gross structures that make standard techniques, such as those applied in the field of cytology, invalid because most of them are sensitive to the presence of noise, and often restricted to the geometric appearance of the cells. In addition, the boundaries of the cell nuclei usually appear blurred, and the fuzzy transition of the boundary between the nuclei and the surrounding background makes the segmentation process a challenging task. 

Over the last decades, the availability of advanced image analysis techniques and software applications, mostly provided from the more theoretically oriented groups in the field of computer vision, has made the progress in the area of histological image analysis more rapid. Early studies on image analysis of tissue sections concentrated primarily on the application of thresholding for image segmentation [7]. Recent studies have leveraged the knowledge gained from low level segmentation to develop more advanced algorithms based on stochastic processes [8], ad hoc image filters [9], and pattern recognition techniques [10]. When prior information about the properties, either color or geometric, of the cellular objects is known, supervised algorithms have been applied for image classification, such as artificial neural networks, boosting approaches (e.g., AdaBoost [11]), and decision trees. For example, in [12] a methodology has been proposed for the segmentation of chromosomes from microscopic images using color features. In [13], a broad set of candidate features has been extracted, using color analysis, template matching, texture analysis, frequency domain techniques, and surface modeling, for classifying lymph node cancers. Without a set of labeled samples, unsupervised techniques, such as fuzzy c-means [14] and self-organizing map [15], have been applied to either classify image regions to different histological structures or identify the magnitude of the lesion in tissue section images [16, 17].

Kostopoulos et al. [18] have shown an important correlation between grading and estrogen receptor status. Grade prediction accuracy was 92.8% relying on a nuclei-to-nuclei basis analysis at high magnifications (>400x), in contrast to the current study that sheds light on the grading problem from a completely different perspective, that is, from the perspective of a frame-to-frame texture analysis at low magnifications (×100). In another study by Tuczek et al. [19], a significant correlation was found between morphological nuclear features (area, perimeter, and diameter) and the grade of each case with *r* = .72. Albert et al. [20] have developed an image analysis system for nuclear grading of breast cancer patients by dividing nuclei into low and high risk groups, with accuracy 88% and 83%, respectively. Aside from histological material, such as the material used in this study, efforts have been made to predict the grade of breast tumors using Fine Needle Aspiration (FNA) cytological material. Such an effort has been presented by Jeleń et al. [21], using a Support Vector Machine pattern recognition system, which was optimized at 94.24% prediction accuracy. Another study based on FNA material by Wolberg et al. [22] has reported a 97% accuracy using an internal 10-fold cross-validation method. A comprehensive review regarding machine learning methods applied for breast cancer diagnosis can be found by Osareh and Shadgar [23].

The aim of this study was to investigate the classification accuracy of three different pattern recognition techniques in the characterization of breast cancer images of tissues sections into different grades of malignancy (grades I–III). In contrast to most other studies that analyze clusters of cells based on high magnification images [18, 24, 25], we employ ×100 magnification as applied in routine clinical practice for deriving a diagnostic grade based on the architecture of the tissue section. Prior to classification, several textural features were extracted from each histological image based on a statistical analysis of the pixel correlations. The performance of each classifier was compared to one another after deriving the optimum combination of the image features. The best classifier was able to generate an overall classification accuracy close to 90%.

The main differences and add-on values of this study in comparison with previous similar studies can be found as follows: (a) frame-to-frame texture analysis at low magnifications (×100) is investigated in contrast to other studies that focus on each nucleus morphological and textural appearance [18, 19], (b) routinely hematoxylin and eosin stained material is used in contrast to FNA cytological material [21, 22], and (c) prediction accuracy is obtained using an external cross-validation method that may be used to estimate the generalization performance of the system to unknown data, in contrast to internal methods that are implied in other studies that might introduce a bias in the classification results [19, 21–23].

## 2. Materials and Methods

The study considered tissue samples from breast cancer biopsies stained with hematoxylin and eosin. The samples were taken from the archives of the Department of Pathology of the Elena Venizelou hospital, Athens, Greece. An experienced histopathologist examined the tissue sections for characterizing the histological tumor grade (I, II, or III). At least 2 representative color images of the lesion were captured from each sample. The images were digitized with x10 magnification using an Olympus BX40F light microscope equipped with an Olympus DP21 digital camera. Based on this magnification, the structure, architecture, and texture of each tissue sample were clearly visible.

**Figure 1:**
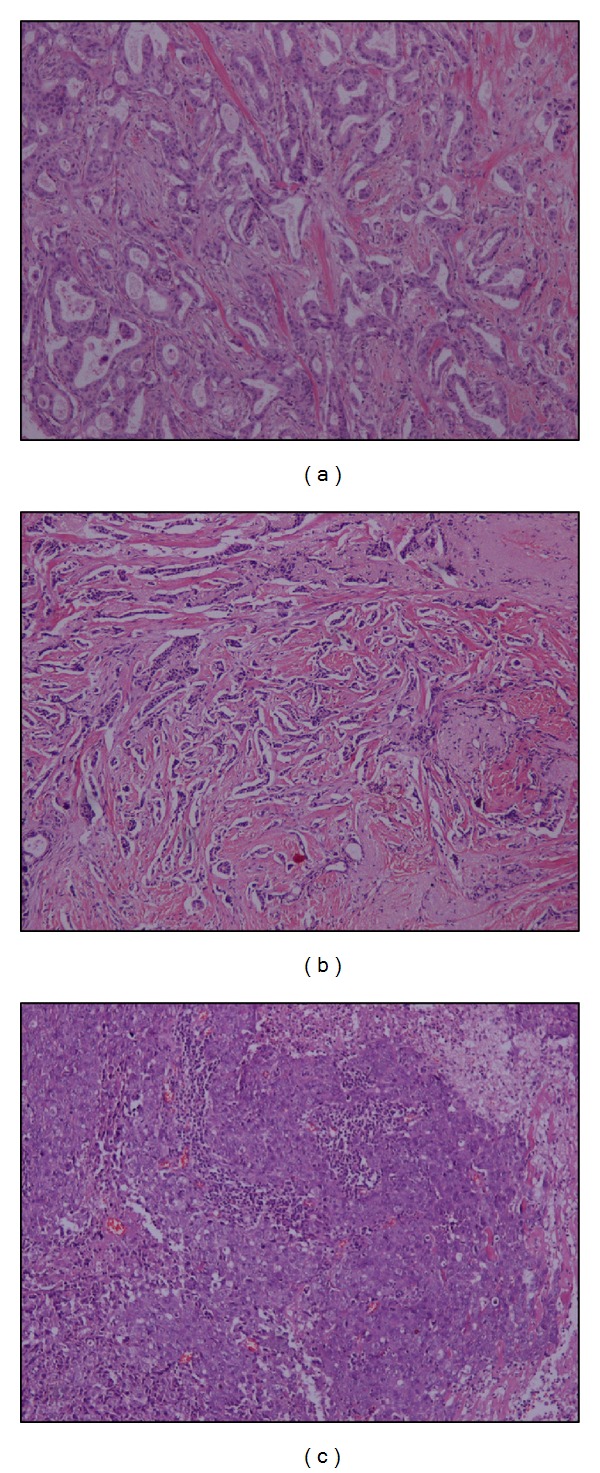
Images of hematoxylin and eosin stained breast biopsies diagnosed as (a) grade I, (b) grade II, and (c) grade III.

The histological dataset included 13 sections originating from an equal number of patients. Five 512 × 512 grayscale regions of interest (ROI) were further extracted from a characteristically diagnostic area of each section collected. The final image dataset included 65 ROIs: 20 grade I, 20 grade II, and 25 grade III. Examples of the three histological classes are shown in Figure 1. 

**Figure 2:**
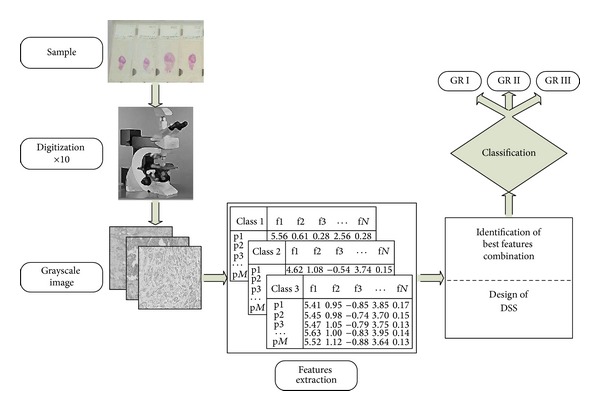
Block diagram of the proposed image analysis system.

A block diagram of the proposed image analysis system is shown in Figure 2. For each ROI, the grayscale intensity channel was considered for further processing and analysis. From each ROI, 30 textural features were extracted in order to design and evaluate a pattern recognition system able to classify the ROI, and consequently the breast cancer cases, into the three histological grades. These features included: 4 first order statistics (mean value, standard deviation, skewness, and kurtosis), 16 second order textural features based on the co-occurrence matrix [26] and 10 based on run-length matrix [27]. 

The classification of breast cancer images to the three malignancy grades was performed using three well-studied classifiers: k-nearest neighbor (kNN) [28], probabilistic neural network (PNN) [29], and support vector machines (SVM) [30]. 

Feature selection was performed by means of the multivariate analysis of variance (manova) statistical test [31], in order to reduce data dimensionality. Features showing statistically significant differences (*P* < 0.001) were further included in the design of the pattern recognition system.

The combination of the best features was determined by employing an exhaustive search [30]: the system was trained and evaluated using feature vectors that comprised all possible feature combinations. Each individual classifier's performance was evaluated by employing the leave-one-out (LOO) method [30]. Based on this method, each classifier was trained with all but one case, which was considered as unknown and was classified to one of the three classes (i.e., grade I–III).

System's generalization performance to unseen data was evaluated based on the external cross-validation (ECV) method [32], where two-thirds of the images were used for system design (finding the best feature combination by LOO method) and the remaining proportion for system evaluation.

## 3. Results and Discussion

Table 1 shows the classification accuracies, partial and overall, of the classifiers and the corresponding feature sets after statistical feature reduction, exhaustive search feature selection, and leave-one-out evaluation.

The discrimination efficiency of the kNN classifier, incorporating three neighbors, was 96.9% since two grade II were misclassified as grade I and grade III. The best combination of the features included the SREa (short run emphasis averaged in four directions), the GLNUa (gray level nonuniformity averaged in four directions), and the RLNUa and the RLNUr (run length nonuniformity averaged and ranged in four directions, resp.). The PNN classifier scored 95.4%, misclassifying one grade II image as grade III and two grade III images as grade II. The best features' combination of the PNN classifier was the SREa, the GLNUa, and the RLNUa. SVM classifier achieved the highest accuracy (96.9%) with the minimum number of features, the SREa and the RLNUa. SVM misclassified one grade II image as grade I and one grade III image as grade II.

Using the ECV method, the whole dataset was randomly split into 10 blocks (training and tests sets) in order to assess the generalization performance of the classification system. kNN and SVM yielded an average accuracy of 85.5% and 84.7%, respectively. The PNN classifier achieved higher mean overall accuracy with smaller standard deviation (89.5 ± 4.4%).  Table 2 shows the partial and overall classification accuracies in ten splits of the dataset and the number of features participated in the best features combination.

**Figure 3:**
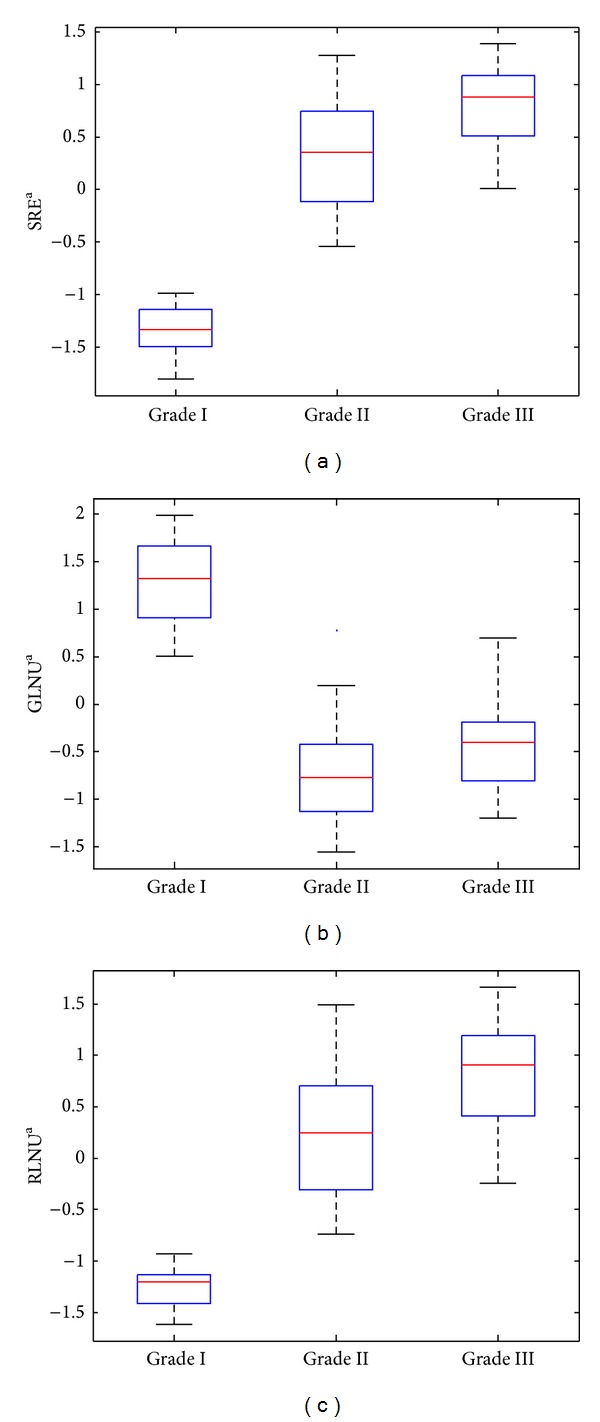
Box plots of short run emphasis, gray level nonuniformity, and run length nonuniformity features for the three histological grades. The horizontal line within each box represents median scores.

Figures 3(a), 3(b), and 3(c) show the box plots of the SRE, GLNU, and RLNU features for the three histological grades. The short run emphasis (SRE) encodes the presence of nuclei and necrosis. Both nuclei and necrosis appeared as small and homogenous structures when low magnification setup was employed. As the histological grade increases, the SRE takes larger values, since the cellularity tends to grow and more nuclei appeared in the same area. The gray level non-uniformity (GLNU) is a measure of structural inhomogeneity in the image and it takes higher values when various structures in the image appeared with similar gray levels. Structures such as the alveolar structures, which are predominant in low histological grade, are clearly visible in low magnification conditions as areas with homogenous gray levels. Thus, in the present work, the GLNU took larger values in low histological grades. The run length nonuniformity (RLNU) is another measure of structural inhomogeneity within the image, and it takes high values when structures with inhomogeneous distribution of runs exist. In the present study, where low magnification was used, the RLNU encoded the information regarding structures such as necrosis, inflammation and stripes. Those structures are mainly occurring when the histological grade increases, and on the presence of these structures the RLNU took higher values.

The good separability of low histological grade images is mainly due to the fact that those images are rich in multiple alveoli and lack necrosis, cellularity, and inflammation that are predominant in higher histological grade images. The previous differences provoke a significant change in the image texture between low and high grade cases. This texture alteration was captured by the textural features selected and might explain the good separability of grade I class.

In general, the small dataset size may introduce a bias in the training stage of a classification system, and this is the reason that the external cross validation method why used. The external cross-validation is suitable when the sample size is relatively small, enabling a fair estimation of the generalization performance of the system to unknown data.

## 4. Conclusions

In this study, the problem of identifying the histological grade of breast cancer tissue sections based on pattern classification and image analysis algorithms was investigated. The main contribution of this research work has emanated from the requirement to develop a robust method for histological grade classification using tissue section images of low magnification. The employment of image-derived textural features that describe the spatial correlations of the grayscale pixels on the image proved a promising approach for the quantification of the architectural pattern of the lesion, and consequently, for the identification of the degree of malignancy (i.e., grade) of the lesion. Image analysis and pattern recognition methods have been previously proposed for the classification of histopathological images of breast cancers, but have been rather focused on textural, morphological, and/or architectural features extracted from the cell nuclei [16–20]. These features are typically viewable in ×40 magnification. In this study, lower magnification images (×10) were deliberately employed in order to investigate whether the inclusion of other important structures, such as necrosis, lymphocytes, inflammation, and adenosis, could provide valuable information about the degree of the malignancy, based on an image analysis framework. The textural features selected, in combination with the pattern classification system, provided promising results with up to approximately 90% mean classification accuracy to unseen data of different malignancy grades. Future extensions of this study will aim towards the investigation of the combination of feature extraction and pattern classification methods on breast cancer images obtained at both low and high magnifications in order to assess potential improvements in the classification accuracy and to obtain a more comprehensive characterization of the tumor malignancy.

## Figures and Tables

**Table 1:** Partial and overall classification accuracies of individual classifiers and leave-one-out method.

	Accuracies (%)				Best features combination
	Grade I	Grade II	Grade III	Overall	
kNN	100	90	100	96.9	SRE^a^, GLNU^a^, RLNU^a^, and RLNU^r^
PNN	100	95	92	95.4	SRE^a^, GLNU^a^, and RLNU^a^
SVM	100	95	96	96.9	SRE^a^, RLNU^a^

SRE: short run emphasis; GLNU: gray level nonuniformity; RLNU: run length nonuniformity; a: average; r: range.

**Table 2:** Partial and overall classification accuracies achieved by employing the PNN classifier and the ECV method.

Trials	Grade I%	Grade II%	Grade III%	Overall accuracy% (no. features)
1	100	83.3	87.5	90.0 (3)
2	100	83.3	75.0	85.0 (2)
3	100	100.0	87.5	95.0 (2)
4	100	83.3	87.5	90.0 (3)
5	100	66.7	87.5	85.0 (3)
6	100	50.0	100	85.0 (3)
7	100	100	87.5	95.0 (3)
8	100	100	75.0	90.0 (2)
9	100	83.3	75.0	85.0 (3)
10	100	83.3	100	95.0 (3)

Mean ± std	100 ± 0	83.3 ± 15.7	86.3 ± 9.2	89.5 ± 4.4

std: standard deviation.
